# Impact of Fat Replacement by Using Organic-Candelilla-Wax-Based Oleogels on the Physicochemical and Sensorial Properties of a Model Cookie

**DOI:** 10.3390/gels9080636

**Published:** 2023-08-08

**Authors:** Cassandra Lizeth Flores-García, Nancy Medina-Herrera, Beatriz Adriana Rodríguez-Romero, Guillermo Cristian Guadalupe Martínez-Ávila, Romeo Rojas, Zahidd Meza-Carranco

**Affiliations:** Chemistry and Biochemistry Laboratory, School of Agronomy, Autonomous University of Nuevo León, Av. Francisco Villa S/N, Col. Ex Hacienda el Canadá, General Escobedo 66050, Nuevo León, Mexico; cassandra.floresga@uanl.edu.mx (C.L.F.-G.); nancy.medinahr@uanl.edu.mx (N.M.-H.); beatriz.rodriguezrm@uanl.edu.mx (B.A.R.-R.); zahidd.mezaca@uanl.edu.mx (Z.M.-C.)

**Keywords:** organic candelilla wax, linseed oil, oleogelation process, physicochemical properties

## Abstract

Oleogelation is an alternative process to improve the nutritional properties of food by creating soft-matter structures with the same functionality as commercial fats (shortenings). In this study, oleogels were produced by adding organic candelilla wax at 3% (OC03), 6% (OC06), and 9% (OC09) to extra-virgin linseed oil, and then characterized by their physicochemical properties. Furthermore, the physicochemical and sensorial properties of five cookie formulations were evaluated. Organic candelilla wax influenced the oleogel formulations, giving higher values of color (L* and b*), texture, acidity index, and melting point. In the cookie formulations, the luminosity values decreased when the percentage of oleogel was increased; reddish trends were obtained (a* values) for the cookie where 70% of the fat was replaced by the oleogel (C70), and more yellow trends were obtained (b* values) for C100. The moisture content was higher in cookies with oleogels, but it was within quality limits. The percentage of fat migration was lower in cookies with a mixture of fats and oleogels. In terms of hardness, the substitution of oleogels resulted in softer cookies. In terms of the sensory evaluation, the most accepted cookie was C70. Therefore, this study demonstrates the possibility of using organic-candelilla-wax-based oleogels in a real food model rich in unsaturated fats.

## 1. Introduction

At present, more countries have established legislative limits to improve food nutritional properties and reduce components related to human health problems, such as trans fats and saturated fatty acids (SFAs) [1]. These legislative limits are because the fat used in most bakery products has a low nutritional value due to its high SFA content. According to WHO recommendations, the daily consumption of SFA must not exceed 10% [2], as the growing consumption of saturated and trans unsaturated fats has been associated with cardiovascular diseases, type II diabetes, and high cholesterol, among other degenerative diseases [3]. However, fat is essential in forming three-dimensional gluten structures in bakery products. The gluten network’s desired strength depends on the formulated product type. In this study, a strong gluten network was not preferred because the cookie prepared is classified as a short dough product. This type of cookie is made with limited water and a large amount of shortening. Shortening is a crucial ingredient in cookie dough because, during mixing, fat crystals form a barrier film around the gluten strands and prevent extensive cross-linking in the gluten network. Likewise, in a cookie dough system, if the fat is poorly distributed throughout the flour particles, they will remain accessible to water for gluten formation [4].

Oleogelation is a strategy to reduce health problems derived from the use of saturated fats. Additionally, oleogels tend to present similar rheological behavior in comparison with SFAs. Oleogels are molecular gels formed by self-assembled supramolecular structures that create a bicontinuous network system on a colloidal scale, in which vegetable oil is trapped by a three-dimensional gelator network [3]. Oleogels have also been used as controlled release systems for lipophilic bioactive compounds, increasing the bioavailability of fat-soluble molecules in functional foods and nutraceuticals [1]. Some researchers have used vegetable waxes to create oleogels, indicating that approximately 3–6% candelilla wax, 5–15% carnauba wax, and 5–10% beeswax are needed to produce stable semisolid structures [5]. Chemically, waxes of vegetable origin are a mixture of long-chain fatty acids, esters of aliphatic alcohols, and hydrocarbons [6] and are used today for several food applications [7], including in the food industry.

In this line, candelilla wax has emerged as a promising potential substance for use in the development of food formulations. The Food and Drug Administration (FDA) ranks this wax as a Generally Recognized as Safe (GRAS) ingredient for its application in the food industry. Candelilla wax is obtained from *Euphorbia antisyphifilitica* Zucc. and is insoluble in water but highly soluble in organic solvents. It is characterized by a high hydrocarbon content (around 50%) and a relatively low number of volatile esters [6]. The use of candelilla wax dates from 1914 and is associated with a simple extraction method. The process begins with collecting the plant, which is placed in cauldrons called “pailas” with a sulfuric acid solution at a concentration of approximately 0.3% (*v*/*v*). In refining, the wax is boiled with sulfuric acid and then water to remove undesirable compounds. However, this practice deteriorates workers’ health due to the generation of toxic fumes. Considering that candelilla wax generated profits of USD 4.9 million from the sale of 1500 tons in 2018 [8], another more suitable method for extracting candelilla wax could be implemented by “candelilleros” (people in charge of extracting candelilla) with some technical, environmental, and health advantages. This alternative process involves the use of organic acids that generate fewer toxic vapors, as has been reported in the extraction of wax using a boiling aqueous solution of citric acid [9]. Moreover, this extraction process can be used to obtain a candelilla wax with characteristics more appropriate for food application, as it causes lower oxidation of its lipidic components compared to the wax extracted via the traditional method [10]. Therefore, the wax obtained via this method can be considered an organic product, since the term “organic” refers to natural and healthy products obtained as a result of agricultural processes formulated with the health of consumers and the conservation of the environment in mind [11]. However, special attention is needed to avoid the overexploitation of this natural resource [8,12].

Previously, several authors have used candelilla wax to confer gelling properties to foods. It has been reported that some structural arrangements occur during oleogel formation due to the presence of self-assembled molecules, crystalline structures, or polymers through the physical modification of the oil entrapping the liquid oil and limiting its flowability [13]. Thus, recent studies demonstrated that food products with modified composition resulting from fat replacement by using different candelilla-wax-based oleogel formulations led to different improved technological parameters in various food models, including a chocolate butter substitute, meat, and bakery products [13,14,15]. Moreover, according to Cabrera et al. [16], oils rich in unsaturated fatty acids result in stronger gels, which could provide more health benefits. In this sense, linseed oil is an adequate option for oleogel formulation because it contains polyunsaturated fatty acids, such as linoleic, linolenic, and γ-linolenic acids [17]. This is because the fatty acid composition of an oil or fat is an important factor for determining stability and defining the primary application. It is also associated with a detrimental impact on human health because the excessive consumption of saturated fatty acids is known to be related to increased plasma cholesterol and obesity. On the other hand, the consumption of polyunsaturated and monounsaturated fatty acids is recommended to improve the lipid profile with respect to saturated fatty acids [18]. Therefore, the consumption of linseed (*Linum usitatissimum*) is beneficial for human health because its oil content ranges from approximately 36% to 40%, and it has more unsaturated fatty acids (88.9%) than saturated acids (11.01%) [19].

It has been reported that oleogel research has produced acceptable reformulated food products with similar technological and rheological properties to reference products or even products with improved technological functionality [9]. However, there is still a great need to improve oleogelation methods and the application of oleogel in food products. By introducing oleogels as cake ingredients, the levels of saturated fatty acids were reduced from 58% to levels as low as 14–17% [20]. Thus, in this work, the formulation of organic candelilla wax oleogels utilizing extra-virgin linseed oil was evaluated for the substitution of fats in “*hojarasca*” cookies, as they have a high saturated fat content which makes this product a good model for oleogel application. In addition, a search in the Scopus database did not reveal previously published information regarding the use of candelilla wax and linseed oil for the preparation of oleogels or their application and evaluation in a bakery product including this food model.

## 2. Results and Discussion

### 2.1. Oleogel Characterization

#### 2.1.1. Visual Appearance

**Figure 1:**
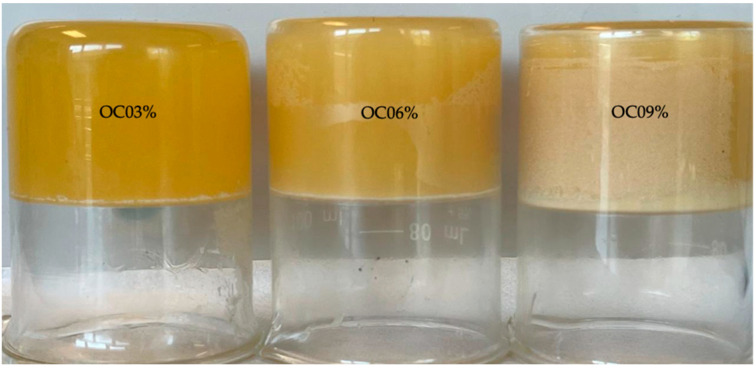
The visual appearance of organic-candelilla-wax-based oleogels.

The appearance of oleogels formulated with extra-virgin linseed oil as an effect of organic candelilla wax concentration is presented in Figure 1. All the oleogels showed a yellowish color with a solid-like texture that did not show visible oil leakage from the structure upon touch. After storage at a temperature of 5 °C, samples with 3% (OC03) organic candelilla wax could be seen as a soft gel that flows over time due to gravity when inverting the glass, but without showing separation of major phases. Samples with 6% (OC06) candelilla wax did not flow when the beaker was inverted; hence, they were identified as stable gels. Furthermore, visually, we found that, compared to the other concentrations, samples with 9% (OC09) candelilla wax formed stronger and more stable gels without alterations when inverting the beaker, indicating that, when the organic candelilla wax concentration increases, more stable oleogels are produced. According to the literature, more stable gels are obtained when a higher concentration (up to 7%) of candelilla wax is used [21]; however, changes in the properties of oleogels can also occur due to external factors such as temperature and other storage conditions. Oleogel stability can be attributed to metastability, which is a well-balanced proportion of the components; however, this stability can be reduced, indicated by the separation of phases and changes from initial physical properties, after periods greater than 12 months [1]. This is also related to storage time, since remarkable stability and no phase separation were observed when 3% wax was used in oleogel formulations after 12 months at 5–25 °C [22].

#### 2.1.2. Physicochemical Characterization

The values for melting point, acidity index, and color obtained in this study are shown in Table 1. There were significant differences among the analyzed samples. The melting points ranged from 37.06 to 61.86 °C. The control samples containing lard (LR) presented the lowest melting point (37.06 ± 1.28), while samples with vegetable shortening (VS) presented a melting point statistically equal to the OC03 samples (47.16 ± 0.41 and 46.23 ± 0.68, respectively). Moreover, higher melting points were obtaining for the oleogel formulations when candelilla wax was increased. The obtained values are similar to those reported by Hwang et al. [23] (except for OC09), who evaluated oleogels formulated with candelilla wax (8%) and different vegetable oils (included linseed oil), which significatively affected the wax crystallization process. The melting properties of waxes are directly related to their chemical components. In addition, the physicochemical characteristics of vegetable waxes depend on several factors, such as the plant species, environmental factors (i.e., water deficit, drought, and geographic area of growth), and age of the plant [9]. Likewise, other waxes, such as sunflower and rice bran wax composed mainly of wax esters (C38 to C54 and C44 to C64, respectively), show high melting points as a result of the wax esters, while candelilla wax contains low-melting-point *n*-alkanes (C29 to C33) and many other low-molecular-weight compounds, which provide a low melting temperature and broad melting peak [23].

In terms of the acidity index, there were significant differences among the analyzed samples. The VS sample presented the lowest value for this parameter, and the LR sample was statistically equal to samples OC03 and OC06; furthermore, the OC09 samples had the highest acidity index value (4.12 ± 1.02 mg KOH/g). This could be related to the increment in free fatty acids present in candelilla [10,24]. However, these values are within the limits set by the CODEX STAN 210-1999 standard for vegetable oils, which establishes the maximum content of free fatty acids for refined oils (0.6 mg KOH/g), virgin cold-pressed oils (4.0 mg KOH/g), and virgin oils (10.0 mg KOH/g) [25].

Color is an important parameter in determining the quality and acceptability of the final product [26]. The values of L*, a*, and b* were statistically different (Table 1) for all the analyzed samples. Only sample OC03 was statistically similar in b* with respect to VS, indicating a tendency to yellow color. Likewise, the increase in L* and b* values was due to a higher concentration of organic candelilla wax in the oleogels used. In the values of the a* parameter, there were differences among the oleogels and both VS and LR; the oleogels presented reddish tendencies (+a*). These findings are similar to those from the report by Merchán-Sandoval et al. [27], who observed an increment in the L* parameter value when the concentration of candelilla wax increased, regardless of the oil used. The used oil had a small effect on a* and b* values. In addition, changes in a* and b* were also due to minor components in oils such as lutein, zeaxanthin (yellow), carotenoids (red), and phospholipids (dark) [28].

Concerning hardness, the puncture test of VS, LR, and oleogels yielded statistically different results (*p* < 0.05) (see Table 1). The oleogel sample where the concentration of organic candelilla wax was 9% (OC09) presented the highest value of hardness among the oleogel samples, followed by OC06 and OC03. With an increase in the concentration of organic candelilla wax, the force required for the puncture test increased throughout the speed interval. Regarding the comparison with lards, oleogel OC03 was statistically equal to lard because vegetable shortening had the highest average (78.40 N), showing a considerably higher value than the oleogels. This is because the hardness of the oleogel also depends on the amount of wax in the oleogel, due to the oleogel hardness increasing with a greater amount of candelilla wax. A similar result was reported by Hwang et al. [23], where they observed a more pronounced increase in oleogel firmness (6.3 N) using 2–10% candelilla wax than in those using linseed oil and other types of oils.

#### 2.1.3. FT-IR Analysis

**Figure 2:**
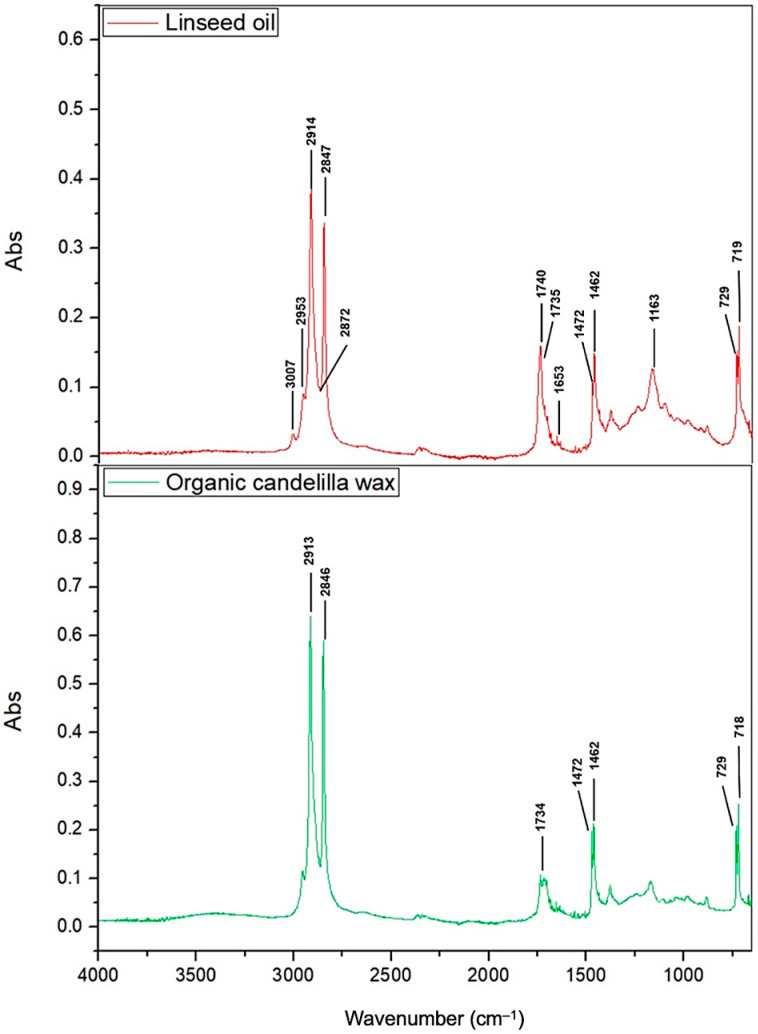
FT-IR spectra of extra-virgin linseed oil (LO) and organic candelilla wax (OCW).

Fourier transform infrared spectroscopy (FT-IR) allowed the identification of the main functional groups present in each oleogel sample. The relevant functional groups encompass hydrogen bonds between sulfonamide and amide groups since they are the main factors in the formation and maintenance of network-shaped structures for the formation of oleogels and are responsible for increasing mechanical resistance by increasing the binding sites of hydrogen [1]. The spectrum of extra-virgin linseed oil is shown in Figure 2. The 3007 cm^−1^ band was assigned to the =C–H symmetric extension of the alkenes (oleofinic bond), while the 2953 cm^−1^ and 2914 cm^−1^ bands were assigned to the asymmetric extensions of CH_3_ and CH_2_. The bands centered at 2872 cm^−1^ and 2847 cm^−1^ can be attributed to the stretching vibrations of CH_3_ and CH_2_ [29] because, in other reports, peaks at 2955, 2915, and 2845 cm^−1^ were attributed to the stretching vibrations (C–H) of the methyl–CH_3_ and methylene–CH_2_ backbones of the n-alkanes [5]. Likewise, the peak at 1740 cm^−1^ was associated with the stretching vibration of the C=O bond of the carbonyl ester group of triglycerides or phospholipids that is characteristic of oils [29], along with the peak at 1735 cm^−1^, which is also associated with C=O stretching [30]. In the 1653 cm^−1^ region, the peak was associated with the stretching vibration of the C=C bond (tiny peak) of vegetable oils [31], corresponding to the degrees of unsaturation of fatty acids. Peaks were also observed at 1472 and 1462 cm^−1^ reflecting the bending of methylene CH_3_ (C–H), while the peak at 1163 cm^−1^ was related to the combination of vibrations of the C–O bond (stretch extension) of the ester group present in the triglyceride molecule and C–H in equilibrium [29,31]. The 729 cm^−1^ and 719 cm^−1^ peaks were associated with the (CH_2_)*^n^* bending of the alchemical chain [32].

The spectrum of the organic candelilla wax is shown in Figure 2. It presented bending at the peaks 2913 cm^−1^ and 2846 cm^−1^, 1472 cm^−1^ and 1462 cm^−1^, and 729 cm^−1^ and 718 cm^−1^. This was due to the C–H stretching vibrations of a saturated carbon showing the existence of a chain of an aliphatic compound with a functional group of long-chain alkanes greater than four carbons in the ranges of 2910–2916 cm^−1^ and 2848–2850 cm^−1^, corresponding to the ester functional group. In the region 1730–1690 cm^−1^, the peak was due to the C=O bond, indicating the presence of a carbonyl group associated with a conjugated aliphatic ester compound [9]. The 1465 cm^−1^ band was ascribed to C–H bending of the *n*-alkanes of the candelilla wax [5] associated with the C–H vibration, indicating the interaction of an aliphatic compound [9].

**Figure 3:**
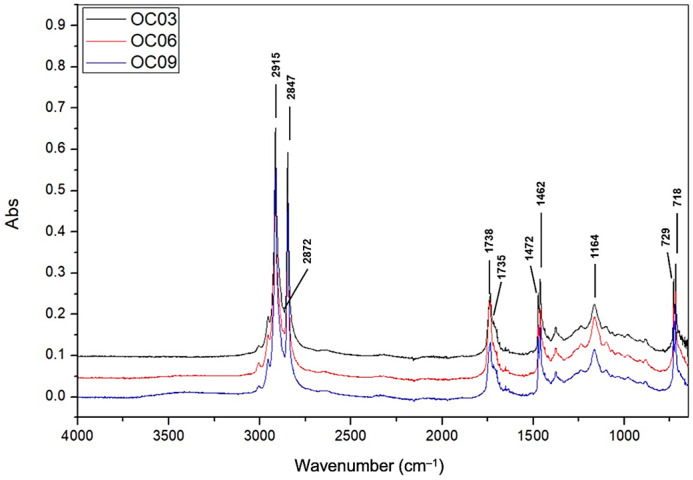
FT-IR spectra of samples: OC03 (3% wax), OC06 (6% wax), and OC09 (9% wax).

**Figure 4:**
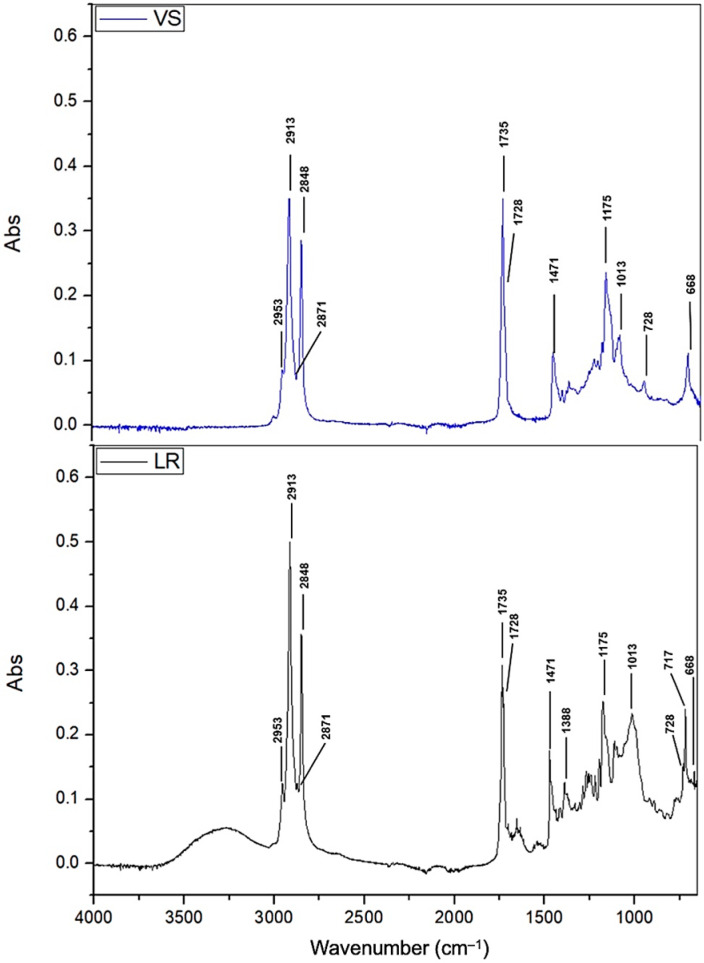
FT-IR spectra of vegetable shortening (VS) and lard (LR).

The spectra of oleogels with candelilla wax and linseed oil presented similar peaks at different intensities (Figure 3). The peaks at 1738 and 1735 cm^−1^ were the result of the oil–wax complex as reported by Trujillo-Ramírez et al. [5] when combining candelilla wax and chia oil, given by the contribution of high-molecular-weight esters of the oil. The decrease in the absorbance in this region in the oleogel samples could be due to the interesterification process [13]. The peaks near the 2925 cm^−1^ band were related to the vibration of the asymmetric section of the C–H bond. Likewise, the peaks near 2855 cm^−1^ were associated with the symmetrical stretching vibration of the C–H bond, and we observed this at 2848 cm^−1^ in lard and 2849 cm^−1^ in vegetable shortening (Figure 4). The peaks at 1739 cm^−1^ for vegetable shortening and 1735 cm^−1^ for lard were related to the stretching of the C=O bond. The peaks at 1463 cm^−1^ for vegetable shortening and 1471 cm^−1^ for lard were related to the scissor vibration of the C–H bond. The peaks at 1172 cm^−1^ for vegetable shortening and 1175 cm^−1^ for lard were associated with the combination of stretching vibrations of the C–O bond and CH rolling. Lastly, those at 719 cm^−1^ for vegetable shortening and 717 cm^−1^ for lard were associated with the balanced vibration of the C–H bond of lipids [31].

### 2.2. Cookies

#### 2.2.1. Physicochemical Properties of Cookies

Color is one of the most important parameters for product acceptance [33]. The results of the parameters L, a*, and b* of the upper part of the cookies are shown in Table 2, showing significant differences among the five samples. For L*, the control cookie sample (CC) was the highest, but without a significant difference from C30. Likewise, the increase in oleogel concentration reduced luminosity. For a*, the highest value was for C70, but there were no statistical differences with C30, C50, and C100. For b*, CC was the sample that presented the lowest value. This might have been due to the replacement of butter with oleogel, which could have reduced lightness [34]. The values of L* at the bottom of the cookie were statistically different among the five samples (*p* ≤ 0.05), while the parameters a* and b* were statistically the same. Other studies reported similar results using other waxes, such as rice bran [28]. These color changes can be attributed to the nonenzymatic browning that occurs with baking, related to Maillard reactions and caramelization of sugars. A higher value of the parameter L* indicates that fats with a higher saturated fat content stick together and block nonenzymatic browning substrates; thus, these reactions do not occur [2]. Due to the addition of oleogel into the cookie formulation, the treatments were statistically different in the upper and lower parts of the cookie (*p* ≤ 0.05). Furthermore, if ΔE > 3, the human eye can observe the color difference; therefore, the detection of the difference by the human eye was possible on the cookie surface [2].

**Figure 5:**
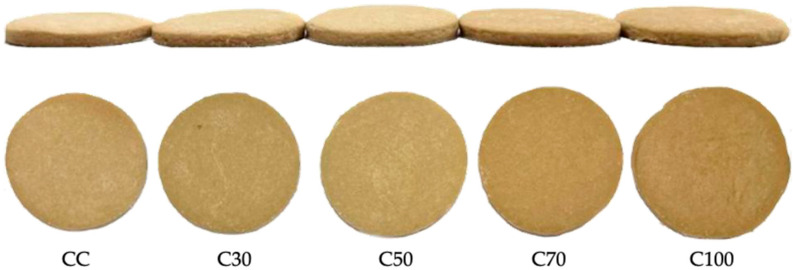
Control cookie (CC), cookie with 30% oleogel (C30), cookie with 50% oleogel (C50), cookie with 70% oleogel (C70), and cookie with 100% oleogel (C100).

Figure 5 shows the cookies with the different variations of the oleogel. The results related to the dimensional characteristics of weight, thickness, diameter, and propagation rate of the cookies were statistically different among the five cookie treatments (Table 3). C50 and C100 were the heaviest cookies, whereas CC and C70 were the lightest.

The thickest cookie was C50, followed by C30. The cookies with the lowest thickness values were those of CC. These changes were due to increased bubbles in the dough during baking due to adding the oleogel [26]. The samples with the largest diameter were C100 and C50, with the smallest values being from CC. This may have been due to the high solid-phase content that improved the retention of gases in the dough structure, resulting in cookies with unsaturated fats having larger diameters than cookies with butter [2]; these findings are similar to those reported by Jang et al. [35]. Likewise, the thickness of the cookies is correlated with a higher moisture content [34].

Furthermore, the highest spread index was presented by sample C70, followed by C100; for the latter, the value was not significatively different from that of CC. The sample C50 obtained a lower propagation index than CC, while C30 was not significatively different. This may be because cookies with a higher solid-phase content have a lower spread index [2]. Likewise, the increase in the incorporation of oleogels is directly proportional to the increase in viscosity, causing a greater force required for the deformation of the dough, and this biaxial extension (viscosity) is related to the propagation speed of the cookies, attributed to the unevenness of the oil content used to formulate the oleogel. Therefore, the percentage of oil in the oleogel causes relatively smaller thicknesses and greater diameters [4].

#### 2.2.2. Moisture Content

There were significant differences in the moisture content. Treatments C50, C70, and C100 had the highest values, which were lower than the maximum limit (15%) according to the regulations [36]. These results are similar to those reported by Mert and Demirkesen [4], who used candelilla wax, but different from those reported by Yilmaz and Öʇütcü [34], who used beeswax and sunflower oil. Therefore, the differences were attributed to the water retention ability of oleogels and shortenings.

#### 2.2.3. Fat Migration Percentage

For the fat migration parameter values, there were significant differences among the analyzed samples, as shown in Table 4. The samples with the highest values were C100 (0.73%) and CC (0.66%), which were significantly equal, while lower values were presented for the samples C50 (0.29%), C30 (0.28%), and C70 (0.18%), which were significantly similar to each other. These changes might have been due to the fat composition used since conventional fats are approximately 79% vegetable oil and other ingredients, while oleogels are more than 90% vegetable oil [34].

Martins et al. [37] suggested that an oleogel formulation with vegetable oils and some long-chain fatty acids or waxes can generate a nutritionally balanced food that is able to develop important biological functions, as well as help the proper functioning of the brain. Therefore, the incorporation of oily systems with mono- and polyunsaturated fatty acids such as linseed oil may be an option. Furthermore, Anastasiu et al. [17] analyzed seven varieties of linseed in an eight-year period and reported amounts ranging from 15.85% to 23.95%, 15.10% to 17.26%, and 53.06% to 60.52% of C18:1, C18:2, and C18:3, respectively. In this sense, the mono- and polyunsaturated fatty acids referenced have a beneficial effect associated with their consumption; for example, C18:1 is related to the prevention of cardiovascular diseases, and C18:3 and C18:3 constitute the membrane of the cell wall and are essential for the normal functioning of the brain [38]. Thus, oleogels formulated with linseed oil are not only attractive due to the composition of fatty acids present in linseed, but they can also serve as a carrier of fat-soluble compounds, such as β-carotene, turmeric, capsaicin, and phenolic compounds [3].

#### 2.2.4. Hardness

The five samples showed statistical differences, as shown in Table 4. Replacing lard with oleogels made the cookies softer. CC had the highest hardness value, followed by C30, C50, and C70, which were significantly equal to the control, with sample CC100 having the lowest value. Hardness is crucial because it influences the product’s acceptance and purchase, given that a hard cookie is considered low quality and unpleasant for the consumer. Similar behaviors were reported when introducing oleogels into cookies using candelilla wax, beeswax, and sunflower wax [4,30,34]. This could have been due to the fine and β-polymorphic crystals of oleogels that do not resist aeration in cookie dough, as reported by Li et al. [39]. Therefore, the distribution of fat and water in the system significantly affects the physical and chemical properties of the cookie dough. Likewise, when liquid oil is used in a system, tiny oil droplets are easily dispersed throughout the dough during mixing, avoiding the formation of cross-links within the gluten network and reducing hardness [4].

#### 2.2.5. Sensory Evaluation

**Figure 6:**
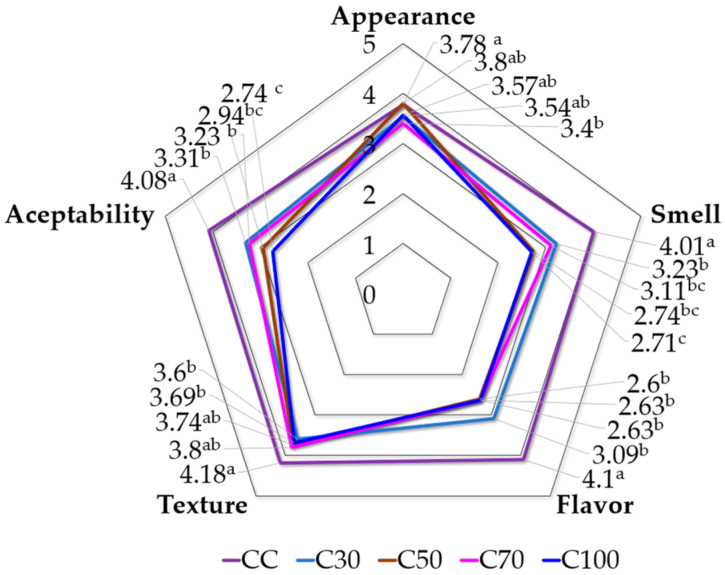
Cookie sensory evaluation: appearance, smell, texture, flavor, and acceptability. Different letters show significant differences according to Tukey’s mean comparison (*p* ≤ 0.05).

The appearance, smell, flavor, texture, and general acceptability during the sensory evaluation statistically differed among the five treatments (Figure 6). The CC, C30, C50, and C100 treatments were statistically equal in terms of appearance, with C70 having the lowest values. Regarding smell, flavor, and texture, CC had the highest values. In the odor parameter considering cookies with oleogel, the highest value was recorded for C30 and the lowest value was recorded for C100 due to the increase in the percentage of oleogel, in agreement with other results reported using beeswax and sunflower and hazelnut oils [33]. In the taste score, the best treatment was C30 and the worst was C50; similar results were reported by Li et al. [39] using beeswax and rice bran wax.

Concerning the texture parameter, the highest value was obtained for G70 and the lowest was obtained for C30, similarly to what was reported by Li et al. [39]. Regarding general acceptability, C30 and C70 had the highest values, above 3.0, giving a good preference level due to the lower level of trans fat and low saturated fat, compared with the lowest level of acceptability for C100 [34]. Lastly, consumers detected a “rancid/bitter”, “vegetable”, or “oily” taste, which may have been due to fat migration. This is associated with the formation of a layer of oil on the product’s surface, which can undergo a faster degradation process outside than inside the product, causing negative sensory properties [2].

## 3. Conclusions

An organic candelilla wax–linseed oil complex allows for the formulation of oleogels. Likewise, using candelilla wax as a substitute for vegetable shortening and lard is a viable option for the formulation of oleogels, influencing their visual appearance, color, hardness, melting point, and acidity index. In addition, it reduces the content of saturated fats in the preparation of cookies. The 9% concentration of candelilla wax (OC09) was the strongest and most stable oleogel, with these properties resulting in statistical differences among the five samples of cookies prepared with a mixture of shortenings (vegetable shortening and lard) and oleogels. Likewise, the increase in the concentration of the oleogel produced more yellow cookies, with C70 being the best treatment. The use of oleogel compared with the control was found to have the same degree of fat migration. However, the C70 treatment was the most accepted by consumers, which is why it is a viable alternative for the almost total substitution of lard or vegetable shortening in cookies. Despite these results, future research efforts should focus on the physical and chemical interactions between organic candelilla wax and the complex assembly of components that exist in other food matrices.

## 4. Materials and Methods

### 4.1. Oleogels

#### 4.1.1. Oleogel Preparation

Oleogel samples were prepared with organic candelilla wax (obtained from Lucio Blanco, Cuatrociénegas, Coahuila, México; coordinates: latitude, 26.165556; longitude, −102.188333) that was blanched and pulverized according to Bernal et al. [40] and mixed with linseed oil (Enature^®^, Jalisco, Mexico) at 3%, 6%, and 9% (*w*/*w*). OC03, OC06, and OC09 were used as sample codes. The mixtures were heated at 100 ± 1 °C, stirring softly for 15 min until the candelilla wax melted completely. The oleogels were left to stand overnight at room temperature for the gelling stage. Finally, the samples were stored at 5 °C in Ziploc bags until use. All treatments were performed in triplicate.

#### 4.1.2. Physicochemical Characterization

The surface color of the oleogels was determined via an instrumental method using a Konica Minolta colorimeter (CR-410, Osaka, Japan). The color parameters L*, a*, and b* were determined by measuring each oleogel’s surface at three different points with an approximate thickness of 10 cm. The L* parameter describes the lightness value, where 0 is black and 100 is white. Negative values of the a* parameter represent green, and positive values represent red colors. Negative values of b* indicate blue, and positive values indicate yellow [2,41].

The acidity index was determined according to NMX-F-101-1987, where 5 g of sample was dissolved with 37.5 mL of ether and 37.5 mL of 95% ethyl alcohol in a water bath. An alcoholic solution of 0.1 N KOH was used with four drops of 1% phenolphthalein [42]. Lastly, for the melting point, the sample was melted in a Petri dish, with a ~1 cm long sample placed in a capillary tube and left to stand at room temperature for 1 day, per Bernal et al. [40].

Hardness testing was performed with a puncture test of the oleogels measured per the modified method by Jang et al. [35]. A Texture Analyzer (TA. XT Plus, Stable Micro Systems, Surrey, UK) with a 5 mm attachment was used for measurement at room temperature (25 ± 2 °C). The probe penetrated the sample up to 10 mm at 1.0 m/s. The maximum force was the measure of hardness recorded from the plot of force versus penetration distance.

#### 4.1.3. FT-IR Analysis

An FT-IR spectrometer coupled with an ATR accessory using a zinc selenide crystal (Cary 630 ZnSe Engine, Agilent, Santa Clara, CA, USA) was used to measure the structural changes in each pure material (organic candelilla wax, linseed oil, and lard) and the oleogels (OC03, OC06, and OC09). The spectra were recorded at 5 °C from 650 to 4000 cm^−1^, with a cycle of 32 scans and a resolution of 2 cm^−1^. The OriginPro program (version 2021) was used to make the graphs.

### 4.2. Cookies

#### 4.2.1. Cookie Preparation

Five different types of cookies were prepared using the formulation in Table 5. Wheat flour (Selecta, Nuevo Léon, México), vegetable shortening (Inca, Ciudad de México, Mexico), lard (Alanis, Coahuila, México), brown sugar (HEB), water, baking powder (REXAL), and 9% candelilla wax oleogel (OC09) (to increase the visual appearance, color, hardness, acidity index, and melting point) were used. The used sample codes were as follows: control with vegetable shortening and lard (CC), cookie with substitution of 30% oleogel (C30), cookie with substitution of 50% oleogel (C50), cookie with substitution of 70% oleogel (C70), and cookie with substitution of 100% oleogel (C100). All ingredients were homogenized in a bowl with a kitchen mixer (Miami, FL, USA) for 13 min at speed 4. The cookie dough was spread to a thickness of 8.5 mm, and 6 cm diameter discs were cut using a circular mold. The cookies were placed on a tray and baked in a gas oven (B00383, Century, Ciudad de México, México) at 200 °C for 25 min. After cooling to room temperature, the cookie samples were packed in polypropylene bags and stored at room temperature until analysis. Two separate batches of cookie production for each of the five types of cookies were made.

#### 4.2.2. Physicochemical Properties of Cookies

The physicochemical properties of the cookies were measured the next day after being baked. The top surface color of the cookie samples was analyzed at three points using a Konica Minolta colorimeter (CR-200, Osaka, Japan), according to the report by Onacik-Gür et al. [2]. The color difference (ΔE) between the cookies with oleogels and the control sample was calculated using Equation (1), where subindex “c” refers to the control, and “m” refers to the sample.

(1) $$\Delta E=\sqrt{{\left(L_{c}^{*}-L_{m}^{*}\right)}^{2}+{\left(a_{c}^{*}-a_{m}^{*}\right)}^{2}+{\left(b_{c}^{*}-b_{m}^{*}\right)}^{2}}.$$

The weight (g), thickness (T), diameter (D), and propagation index (PI) of the four samples of randomly selected cookies were recorded using an analytical balance (Edeardda, BMS220.4, Washington, WA, USA) and a digital vernier (2ZA61, Westward, China). Four measurements were made for each cookie, and their averages were reported. According to the report [35], the propagation index (PI = D/E) was calculated by dividing the diameter by thickness. The moisture content of the samples was determined gravimetrically with the same analytical balance according to the report by Mert and Demirkesen [4], recording four measurements for each cookie.

#### 4.2.3. Determination of Fat Migration

Fat migration was determined according to the report by Onacik-Gür and Żbikowska [2] with slight modifications. Cookies were placed in an oven (ON-12G, JEIO TECH, Seoul, Korea) preheated to 30 °C on five layers of paper (120 g/m^2^) on Petri dishes. After 24 h, the weight of the paper on the dish was recorded. The fat migration was calculated on the basis of the difference in mass (g). The assay was performed in quadruplicate.

#### 4.2.4. Hardness

Cookie hardness was determined according to the reported method by Onacik-Gür and Żbikowska, Yilmaz, and Öʇütcü [2,34] with some modifications, considering the breaking force using a compression attachment of one point of 2 mm on a perforated base. The compression method was selected using a Texture Analyzer (TA. XT Plus, Stable Micro Systems, Surrey, UK) with a velocity of 3.00 mm/s and 7.00 mm of distance. The probe was removed at a 0.5 mm/s velocity with a firing force of 0.04930 N. The maximum peak force required to break the cookies was recorded as cookie hardness. A cookie was placed on the perforated base and the probe was removed at a 0.5 mm/s velocity with a firing force of 0.04930 N. The maximum peak force required to break the cookies was recorded as cookie hardness. Three samples were used, and the average was reported.

#### 4.2.5. Sensory Evaluation

Regarding sensory evaluation, the consumer hedonic scores of the cookies were used on a five-point scale (from 1 for dislike very much to 5 for like very much) to assess sensory attributes such as appearance, texture, flavor, smell, and acceptability. The analysis was performed in two sessions, the first with 35 consumers (24 women and 11 men, aged 17 to 67 years old) and the second with 35 other consumers (21 women and 14 men, aged 17 to 35 years old); both sessions were presented with the control code in random order. Each panelist was provided with water to clean their oral cavity between tastings.

### 4.3. Statistical Analysis

To determine the statistical differences, an ANOVA test was performed, followed by Tukey’s test to compare mean values. Minitab software (2019 Version) was used to find statistical differences between treatments at a 95% confidence level (α = 0.05). The means were compared with Tukey’s test (α = 0.05).

## Figures and Tables

**Table 1:** Color, hardness, melting point, and acidity index of vegetable shortening (VS), lard (LR), and oleogels (OC03, OC06, and OC09).

Sample		Color		Hardness (N)	Melting Point (°C)	Acidity Index (mg KOH/g)
	L*	a*	b*			
VS	72.02 ± 1.65 ^b^	2.54 ± 0.12 ^a^	13.75 ± 0.44 ^c^	78.40 ± 4.01 ^a^	47.16 ± 0.41 ^c^	0.18 ± 0.02 ^c^
LR	88.32 ± 0.22 ^a^	0.67 ± 0.13 ^b^	7.65 ± 0.20 ^d^	1.46 ± 0.18 ^c^	37.06 ± 1.28 ^d^	1.93 ± 0.05 ^b^
OC03	42.53 ± 1.72 ^e^	−1.64 ± 0.60 ^cd^	16.19 ± 1.93 ^c^	0.38 ± 0.09 ^c^	46.23 ± 0.68 ^c^	1.57 ± 0.48 ^b^
OC06	55.60 ± 1.26 ^d^	−1.72 ± 0.13 ^d^	23.26 ± 0.53 ^b^	3.63 ± 0.06 ^bc^	52.76 ± 1.66 ^b^	2.08 ± 0.05 ^b^
OC09	63.08 ± 1.14 ^c^	−0.89 ± 0.11 ^c^	28.37 ± 1.29 ^a^	6.43 ± 0.27 ^b^	61.86 ± 2.50 ^a^	4.12 ± 1.02 ^a^

Different letters show significant differences according to Tukey’s mean comparison (*p* ≤ 0.05).

**Table 2:** Colorimetry results on the upper and bottom parts of the cookies.

Sample	Top Color				Bottom Color			
	L	a*	b*	ΔE	L	a*	b*	ΔE
CC	62.24 ± 1.08 ^a^	7.26 ± 0.58 ^b^	22.65 ± 0.58 ^b^	0.00 ± 0.00 ^d^	60.58 ± 2.08 ^a^	9.44 ± 1.01 ^a^	24.71 ± 1.17 ^a^	0.00 ± 0.00 ^b^
G30	61.58 ± 1.10 ^a^	7.47 ± 0.42 ^ab^	23.65 ± 0.46 ^ab^	1.83 ± 0.28 ^cd^	58.28 ± 0.87 ^ab^	10.33 ± 0.75 ^a^	26.49 ± 0.92 ^a^	3.05 ± 2.28 ^b^
G50	59.20 ± 0.77 ^b^	7.88 ± 0.23 ^ab^	23.32 ± 0.31 ^ab^	3.21 ± 1.25 ^bc^	55.15 ± 0.60 ^ab^	10.90 ± 0.58 ^a^	27.80 ± 0.92 ^a^	6.43 ± 1.92 ^a^
G70	58.02 ± 0.75 ^bc^	8.25 ± 0.30 ^a^	23.90 ± 0.22 ^a^	4.60 ± 1.67 ^b^	55.27 ± 0.92 ^ab^	10.88 ± 0.77 ^a^	27.44 ± 1.14 ^a^	6.75 ± 2.13 ^a^
G100	56.36 ± 0.88 ^c^	8.00 ± 0.36 ^ab^	24.07 ± 0.75 ^a^	6.28 ± 1.15 ^a^	54.25 ± 3.80 ^b^	9.71 ± 2.63 ^a^	24.37 ± 2.97 ^a^	7.22 ± 4.87 ^a^

Different letters show significant differences according to Tukey’s mean comparison (*p* ≤ 0.05).

**Table 3:** Dimensions of the cookies in weight, thickness (T), diameter (D), and spread index (SI).

Sample	Weight (g)	T (mm)	D (mm)	SI (D/T)
CC	14.47 ± 0.55 ^c^	7.75 ± 0.51 ^b^	58.90 ± 0.44 ^b^	7.61 ± 0.19 ^ab^
C30	17.60 ± 1.74 ^bc^	8.90 ± 0.95 ^ab^	60.59 ± 0.78 ^ab^	6.87 ± 0.61 ^bc^
C50	22.54 ± 2.37 ^a^	10.40 ± 0.80 ^a^	62.68 ± 1.64 ^a^	6.04 ± 0.19 ^c^
C70	14.82 ± 0.85 ^c^	7.02 ± 0.57 ^b^	61.14 ± 0.65 ^ab^	8.75 ± 0.68 ^a^
C100	20.09 ± 1.92 ^ab^	8.47 ± 0.44 ^b^	63.33 ± 1.58 ^a^	7.48 ± 0.15 ^abc^

Different letters show significant differences according to Tukey’s mean comparison (*p* ≤ 0.05).

**Table 4:** Results of the percentage of moisture content (MC), fat migration (FM), and hardness of the cookies.

	CC	C30	C50	C70	C100
MC (%)	2.55 ± 0.65 ^b^	2.93 ± 0.13 ^b^	4.45 ± 0.29 ^a^	4.27 ± 0.80 ^a^	4.29 ± 0.20 ^a^
FM (%)	0.66 ± 0.21 ^a^	0.28 ± 0.17 ^b^	0.29 ± 0.09 ^b^	0.18 ± 0.09 ^b^	0.73 ± 0.15 ^a^
Hardness (N)	7.73 ± 2.90 ^a^	4.83 ± 0.59 ^ab^	4.82 ± 0.92 ^ab^	5.03 ± 1.28 ^ab^	4.48 ± 0.99 ^b^

Different letters show significant differences according to Tukey’s mean comparison (*p* ≤ 0.05).

**Table 5:** The formulation of cookies in percentages.

Ingredients	%	%	%	%	%
	CC	C30	C50	C70	C100
Wheat flour	52.6	52.6	52.6	52.6	52.6
Vegetable shortening	18.7	13.1	9.4	5.6	0.0
Lard	6.6	4.6	3.3	2.0	0.0
Sugar	12.8	12.8	12.8	12.8	12.8
Water	8.8	8.8	8.8	8.8	8.8
Baking powder	0.6	0.6	0.6	0.6	0.6
(Linseed oil)	0.0	6.9	11.5	16.1	23.0
Organic candelilla wax	0.0	0.7	1.1	1.6	2.3

## Data Availability

The data presented in this study are available on request from the corresponding author. The data are not publicly available due to the student thesis is into finish process.
